# Acute renal failure and neurological manifestations following ingestion of wild mushrooms

**DOI:** 10.4103/0971-4065.59347

**Published:** 2009-10

**Authors:** F. Frantzeskaki, M. Theodorakopoulou, I. Mavrou, A. Armaganidis

**Affiliations:** 2^nd^ Department of Critical Care, Atticon University Hospital, Athens, Greece

Sir,

A 58-year-old nonalcoholic man was admitted to the emergency department with one day history of confusion, vomiting, oliguria. He was under no medication or drug abuse. His blood pressure was 140/80 mmHg, pulse rate 80 bpm, body temperature 38°C, and respiratory rate 30 breaths/min. Physical examination was remarkable for pinpoint pupils (miosis) bilaterally; no signs of meningeal irritation were noticed. The patient was lethargic, he obeyed to doctor's commands and communicated with confused conversation, showing disorientation and disorganized thinking (GCS:14). He presented with a deep and labored breathing pattern, such as Kussmaul breathing. He was treated with ceftriaxone 2 g/day, as empirical therapy for bacterial meningitis. The patient was intubated due to impending coma and acute respiratory failure and was transferred to the intensive care unit (ICU). Laboratory data revealed: WBC: 18000/ cm^3^ with neutrophils 80%; Hct: 38; PLT: 200000; Blood Urea Nitrogen: 110 mg/ dl; Creatinine: 5.2 mg/ dl; Potassium: 4 MEq/l; Sodium: 139 mEq/l; Chloride: 101 mEq/l; SGOT: 19; SGPT: 16; LDH: 459; γGT: 18; total bilirubin: 0.68. Arterial blood gases, under assist control ventilation with FiO_2_ 60%, tidal volume 500 ml και breathing rate 14, were: PO_2_: 268 mmHg, PCO_2_: 31 mmHg, PH: 7.32, HCO_3_: 16. A brain C/T revealed no abnormal signs and a chest C/T showed bibasilar opacities. A lumbar puncture was performed and cerebrospinal fluid (CSF) test results were: White blood cell (WBC): 2/mm^3^ (28% polymorphs, 72% lymphocytes), protein: 65 g/L, glucose: 85 mg/ dl. Cerebrospinal fluid (CSF) Gram's stain and cultures, and tests for HSV 1 and 2, enteroviruses, Coxsackie viruses, Listeria monocytogenes, CMV and Borelia were all negative. Blood cultures, IgG and IgM antibodies for leptospira interrogans, borellia burgdorferi, brucella, coxiella burnetii and immunologic tests for vasculitis were also negative. Renal ultrasound revealed normal-sized kidneys and no hydronephrosis. The patient was placed on continuous venovenous hemodiafiltration (CVVHFD) due to anuric acute renal failure (ARF) of unknown origin. Ceftriaxone 2 g/d and clindamycin (600 mg/td) were provided for presumed aspiration pneumonia. Renal biopsy showed marked focal interstitial lymphocytic infiltrate, consistent with interstitial nephritis. Two days later, the patient was apyrexial and alert and was successfully extubated after a short weaning trial. He acknowledged having consumed wild, brown-coloured mushrooms two days prior to admission. Two friends of his had tried the same mushrooms, without developing any symptoms. The patient was transferred to a dialysis department; his renal function gradually improved and he was discharged four weeks later in an excellent condition.

The medical history and clinical presentation of the patient were consistent with mushroom intoxication. Other causes of interstitial nephritis and altered consciousness such as Wegener's granulomatosis, microscopic polyangiitis, leptospirosis, methanol or salicylate intoxication were excluded. Although the exact type of mushrooms ingested by the patient was not identified, his clinical pattern was similar to intoxication by mushrooms from the genus cortinarius [Figure 1]. This particular type contains the toxin orellanine, which is responsible for acute interstitial nephritis and ARF, while preserving hepatic function.[1] Its oxidation leads to the production of reactive oxygen species and depletion of glutathione, rendering renal cells susceptible to oxidant damage.[2]

**Figure 1:**
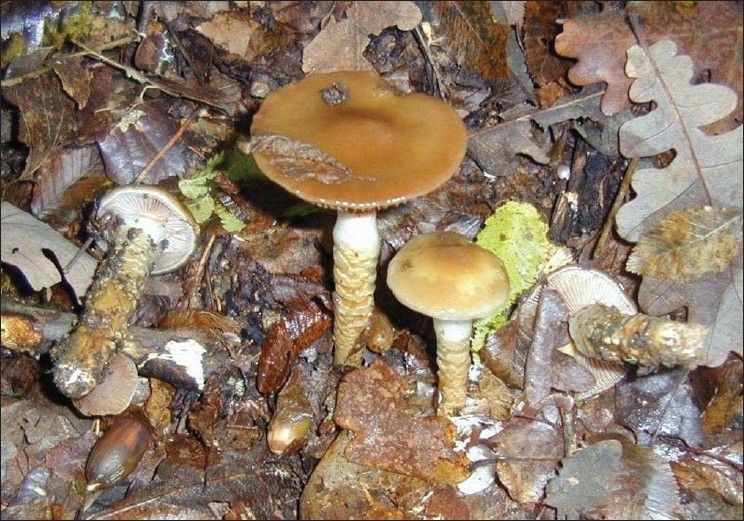
Mushrooms of genous cortinarius

The clinical picture consists of an asymptomatic period, followed by oliguric renal failure 2-20 days postingestion. Common symptoms include nausea and vomiting, neurological manifestations (paraesthesia, cognitive disorders), parasympatheticomimetic reactions (for example, mysis), lumbar and abdominal pains.[1] Wide variations in individual sensitivity are observed and there is no correlation between the amount ingested and the intensity of signs.[3] The high individual sensitivity may suggest that renal toxicity results from the toxin's hepatic metabolism and the hydroxylation capacity of hepatic P450 cytochromes.[4] Additionally, our patient did not mention any use of acetaminophen, which might have depleted glutathione faster and could explain the intoxication. The incidence of renal failure varies from 30-45%,[4] with acute tubulointerstitial nephritis, as the predominant finding in renal biopsy.[1] Recovery of renal function is obtained within a month in 64% of the cases, while in all other cases renal failure persists.[4] Hemodialysis is the only appropriate treatment, which may eliminate the free circulating toxin, while furosemide may aggravate renal lesions.[5] N-acetylcysteine is another reported treatment specific to orellanine intoxication, but its benefit remains debatable.[2,6]

In our patient, the syndrome of unexplained tubulointerstitial nephritis with neurological manifestations was finally attributed to cortinarius poisoning. Clinicians should be aware of these potential toxicities that are characterized by high individual sensitivity and may result in permanent renal lesions.

## References

[1] Mount P, Harris G, Sinclair R, Finlay M, Becker GJ (2002). Acute renal failure following ingestion of wild mushrooms. Intern Med J.

[2] Kilner RG, D'Souza RJ, Oliveira DB, MacPhee IA, Turner DR, Eastwood JB (1999). Acute renal failure from intoxication by Cortinarius orellanus: Recovery using anti-oxidant therapy and steroids. Nephrol Dial Transplant.

[3] Gerault A Intoxication collective de type Orellanien provoquee par Cortinarius Splendens R. Hy. Bull Soc Mycol France.

[4] Bouget J, Bousser J, Pats B, Ramee MP, Chevet D, Rifle G (1990). Acute renal failure following collective intoxication by Cortinarius orellanus. Intensive Care Med.

[5] Nieminen L, Pyy K, Hirsimäki Y (1976). The effect of furosemide on the renal damage induced by toxic mushroom Cortinarius speciosissimus in the rat. Br J Exp Pathol.

[6] Koppel C (1993). Clinical symptomatology and management of mushroom poisoning. Toxicon.

